# Isolated C3 hypocomplementemia as an early predictor of chronic kidney disease in lupus nephritis

**DOI:** 10.3389/fimmu.2025.1655825

**Published:** 2025-09-12

**Authors:** Simeone Andrulli, Lucio Manenti, Francesco Reggiani, Isabella Pisani, Domenico Giannese, Gisella Vischini, Giovanni Valsecchi, Giulia Godeas, Giuseppe Gigliotti, Pasquale Esposito, Paola De Giovanni, Corrado Murtas, Chiara Casuscelli, Sabrina Caruso, Michele Rossini, Giovanni Andrulli, Marco Quaglia, Filippo Aucella, Andrea Buscaroli, Giovanni Maria Rossi, Francesca Mattozzi, Brigida Di Renzo, Fulvia Zanchelli, Francesca Bruno, Raffaela Sciri, Massimo Manes, Diletta Domenica Torres, Maurizio Garozzo, Roberta Lazzarin, Valentina Corbani, Francesco Fontana, Marta Calatroni, Monia Incerti, Claudia Bini, Barbara Infante, Pierluigi D’Angio’, Margherita Di Martino, Angelo Rigotti, Loreto Gesualdo

**Affiliations:** ^1^ Associazione Italiana Ricercare per Curare ODV ETS (AIRpC), Lecco, Italy; ^2^ Nephrology Unit, Azienda Sociosanitaria Liguria 5, La Spezia, Italy; ^3^ Nephrology and Dialysis Unit, IRCCS Humanitas Research Hospital, Rozzano, Italy; ^4^ Nephrology Unit, Parma University Hospital, Parma, Italy; ^5^ Nephrology, Dialysis, Transplantation, Azienda Ospedaliero Universitaria Pisana, Pisa, Italy; ^6^ Nephrology, Dialysis and Kidney Transplant Unit, IRCCS Azienda Ospedaliero-Universitaria di Bologna, Bologna, Italy; ^7^ Nephrology, Dialysis and Transplantation Unit, Department of Biomedical Sciences, Foggia, Italy; ^8^ Nephrology and Dialysis Unit, Maria Santissima Addolorata Hospital, Eboli, Italy; ^9^ UO Nefrologia, Dialisi e Trapianto, IRCCS Policlinico San Martino, Genova, Italy; ^10^ Nephrology and Dialysis Unit, Ospedale Degli Infermi, Rimini, Italy; ^11^ Nephrology and Dialysis Unit, ASL Viterbo, Viterbo, Italy; ^12^ Nephrology and Dialysis Unit, Department of Clinical and Experimental Medicine, AOU “G. Martino”, Messina, Italy; ^13^ Nephrology and Dialysis Unit, ASST Fatebenefratelli-Sacco, Milano, Italy; ^14^ Nephrology, Dialysis and Transplantation Unit, Department of Emergency and Organ Transplantation, University of Bari Aldo Moro, Bari, Italy; ^15^ Bocconi University, Milan, Italy; ^16^ SCDU Nefrologia e Dialisi, AOU “SS Antonio e Biagio e Cesare Arrigo”, Alessandria, Italy; ^17^ Nephrology and Dialysis Unit, “Casa Sollievo Della Sofferenza” Foundation, Scientific Institut for Research and Health Care, San Giovanni Rotondo, Italy; ^18^ Nephrology and Dialysis Unit, Ospedale Santa Maria delle Croci, Ravenna, Italy; ^19^ Nephrology and Dialysis Unit, Regina Margherita, Torino, Italy; ^20^ Nephrology and Dialysis Unit, Ospedale A. Perrino, Brindisi, Italy; ^21^ Nephrology and Dialysis Unit, Morgagni-Pierantoni Hospital, Forlì, Italy; ^22^ Nephrology and Dialysis Unit, Policlinico Gemelli, Roma, Italy; ^23^ Nephrology and Dialysis Unit, S.Maria della Misericordia Hospital, Perugia, Italy; ^24^ Nephrology and Dialysis Unit, Umberto Parini Hospital, Aosta, Italy; ^25^ UOSD Nefrologia e Dialisi Pediatrica, Ospedale Giovanni XXIII, Bari, Italy; ^26^ Nephrology and Dialysis Unit, Santa Marta and Santa Venera Hospital, Acireale, ASP 3 Catania, Acireale, Italy; ^27^ Nephrology and Dialysis Unit, Ospedale San Giacomo Apostolo, Castelfranco Veneto, Italy; ^28^ Nephrology and Dialysis Unit Sant’Andrea Hospital, La Spezia, Italy; ^29^ Nephrology, Dialysis and Kidney Transplant Unit, Azienda Ospedaliero Universitaria di Modena, Modena, Italy

**Keywords:** CKD, ESKD, proteinuria, complement, lupus, immunosuppression

## Abstract

**Objective:**

The role of complement in the long-term renal survival of patients with lupus nephritis (LN) remains poorly understood. Recent studies suggest its potential impact; however, long-term data are lacking.

**Methods:**

This multicenter, observational, retrospective study aimed at investigating the influence of complement levels on long-term renal outcomes in LN patients. We evaluated whether isolated C3 hypocomplementemia (i-LowC3), defined as serum low C3 (≤80 mg/dL) and normal C4 (>10 mg/dL) six months after kidney biopsy is associated with subsequent risk of chronic kidney disease (CKD), End Stage Kidney Disease (ESKD) or death.

**Results:**

445 patients with LN were studied (median follow-up 4.9 years). Based on six-month C3/C4 levels, patients were categorized into i-LowC3 (91 patients) and controls (354 patients). Over the first six months, serum C3 and C4 levels increased by a median of 20 mg/dL and 5 mg/dL, respectively. i-LowC3 was significantly associated with twice the risk of a poor outcome, including CKD, ESKD, composite outcome of CKD or death and ESKD or death, with lower survival rates for all these outcomes compared to controls (P < 0.001). Multivariate Cox regression analysis revealed a lower risk of CKD and CKD or death with increases in C3 levels during the first six months, while i-LowC3 was associated with an independent higher risk for these outcomes.

**Conclusion:**

The trajectory of serum C3 levels within the first six months appears to predict long-term renal prognosis of LN patients. These findings support the use of i-LowC3 as a low-cost, readily available biomarker to guide early treatment of LN patients.

## Introduction

Lupus nephritis (LN) is a major complication of systemic lupus erythematosus (SLE), affecting approximately 30 – 40% of SLE patients. Its onset is typically characterized by proteinuria, alterations in urinary sediment, and, in some cases, acute renal failure (1).

Numerous studies have sought to identify prognostic predictors of End-Stage Kidney Disease (ESKD) in patients with LN to optimize the administration of immunosuppressive therapy according to individual risk profiles. Established prognostic factors include baseline estimated glomerular filtration rate (eGFR), a high National Institutes of Health Chronicity Index, non-Caucasian ethnicity, duration of SLE prior to LN onset, age at diagnosis, and the presence of hypertension (2, 3), which primarily reflect pre-existing, non-specific renal damage rather than identifying a distinct high-risk phenotype. Conversely, an early predictor of favorable long-term renal outcomes in contemporary cohorts is low proteinuria at 12 months (4–6).

Complement proteins are among the earliest and most extensively studied pathogenic mediators in SLE (7–13). Whether complement activation can reliably predict the development of chronic kidney disease (CKD) in LN patients has been a subject of ongoing debate (14–16). Conflicting data on this topic arise partly because complement activation in SLE has dual roles. While overactivation may contribute to tissue injury, the complement classical pathway (cCP) has protective functions, including the clearance of immune complexes, cellular debris, and apoptotic bodies. Low copy number and/or genetic deficiencies in the cCP are well-documented predisposing factors for the development of SLE (17).

Conversely, dysregulation of the complement alternative pathway (cAP) can exacerbate renal injury. Excessive production of potent anaphylatoxins, such as C5a, drives neutrophil recruitment and sustains inflammation during acute renal flares. Persistent overactivation of the cAP further contributes to endothelial damage, promoting the progression from acute kidney injury to CKD (18).

Unlike atypical hemolytic uremic syndrome, which is often characterized by a genetically determined dysfunction of cAP regulatory proteins, no similar genetic pattern has been identified in SLE or LN. However, recent findings suggest that cAP overactivation in LN may be attributed to the presence of circulating anti-C3 antibodies. These autoantibodies, detected in approximately one-third of LN patients, appear to inhibit cAP regulatory factors such as factor H and complement receptor 1, thereby stabilizing the cAP C3 convertase (18, 19).

Moreover, anti-C3 antibody titers are inversely correlated with persistent serum C3 consumption and are significantly associated with inflammatory renal damage in LN patients. These findings provide a potential explanation for the dual role of complement in LN pathogenesis. While the physiological activation of the cCP exerts both inflammatory and protective effects, cAP dysregulation leads to progressive endothelial damage, culminating in thrombotic microangiopathy (20).

In a previous retrospective study, a persistent hyperactivation of the cAP—identified in patients with persistently low serum C3 levels six months after initiating immunosuppressive therapy for a first episode of proliferative LN—was associated with the progression of renal damage and the development of ESKD over the long term (18).

Our study aimed to validate these findings in a large independent Italian cohort of the Italian Registry of Renal Biopsy.

## Materials and methods

### Patient selection

The Italian study centers involved in this observational multi-center study are detailed in the Appendix. As an observational study, patient enrollment criteria were not subject to modification. All consecutive patients undergoing native kidney biopsy during the active recruitment period, with a primary histological diagnosis of LN, were deemed eligible for inclusion.

Exclusion criteria included patients with LN who had a follow-up period of less than three months, those without available C3 and C4 determinations prior to native kidney biopsy, and patients with previously diagnosed LN who underwent repeat kidney biopsy. The latter exclusion aimed at minimizing biases related to prior exposure to immunosuppressive therapies.

Data collection was centralized and conducted through a purpose-built web-based database (http://www.statgate.it) integrated with the Italian Registry of Renal Biopsy (IRRB) (http://www.irrb.net). All patients provided written informed consent, and the study protocol received approval from the Ethics Committee of Bari University. The study adhered to the principles of the Declaration of Helsinki and was conducted as an independent initiative without external sponsorship. It was registered at ClinicalTrials.gov (No. NCT04948593).

### Study groups

The primary objective of this study was to evaluate the long-term renal survival of patients with a primary diagnosis of LN, focusing on the potential independent predictive value of complement C3 and C4 levels at the six-month follow-up after native kidney biopsy. Following a similar approach to our previous study (18), the cohort was divided into two groups: patients with isolated C3 hypocomplementemia (i-LowC3), defined as low serum C3 levels (≤80 mg/dL) and normal serum C4 levels (>10 mg/dL), at six months after the initiation of immunosuppressive therapy, and a control group.

### Study outcomes

Four key outcomes were assessed: (i) the development of CKD, defined as an eGFR) ≤60 mL/min/1.73 m², as calculated using the CKD-EPI equation (21); (ii) the composite outcome of CKD or death; (iii) the development of ESKD; (iv) the composite outcome of ESKD or death.

Additionally, the trajectory of serum C3 levels within the first six months, following immunosuppressive therapy, was tested to predict renal prognosis in patients with LN. The study also evaluated possible confounding variables, including baseline kidney function, age, gender, proteinuria levels before biopsy, proteinuria reduction during the first six months of follow-up, and the number and type of immunosuppressive drugs used.

### Variables

Relevant patient-related covariates recorded included age, gender, eGFR (calculated using the CKD-EPI equation (21)), 24-hour proteinuria magnitude, and its reduction over the first six months of follow-up, and the LN histological class according to the International Society of Nephrology/Renal Pathology Society (ISN/RPS) classification (22).

### Statistical analysis

Quantitative variables were summarized using medians and the 10th and 90th percentiles as measures of central tendency and variability, respectively. The Mann-Whitney U test was employed to compare continuous variables between the two groups defined by C3/C4 levels at six months. Categorical variables were reported as absolute numbers and percentages, and comparisons were conducted using the Chi-squared test.

Kaplan-Meier survival curves were generated for each outcome, stratified by six-month C3 and C4 levels using cut-off values of 80 mg/dL and 10 mg/dL, respectively. Differences in survival between groups were assessed using the log-rank test.

For inferential analysis, multivariate Cox regression was performed for the defined outcomes. Predictor variables were parametrized according to their categorical, discrete, or continuous nature, with indicator dummy variables used for categorical predictors (1 for present, 0 for absent). A stepwise iterative approach was applied, beginning with a full model, followed by a backward elimination strategy using the Likelihood Ratio (LR) test to determine the final model. Inclusion and exclusion criteria for covariates were based on thresholds of Pin = 0.1 and Pout = 0.05. The missing data excluded the corresponding records from the analysis. A P-value ≤0.05 was considered statistically significant. For each selected covariate, beta coefficients, exponential beta coefficients (as estimates of Relative Risk, RR), and their 95% confidence intervals were reported.

All statistical analyses were conducted using the Statistical Package for Social Sciences (SPSS for Windows, version 23.0).

## Results

This study involved 26 Italian centers (detailed in the **Appendix**) and included 609 patients with a histological diagnosis of LN, enrolled between June 15, 1980, and September 11, 2023. After excluding patients with a follow-up period of less than three months (56 patients, 9.2%), those without C3 and C4 measurements before kidney biopsy (17 patients, 2.8%), and patients with repeated native kidney biopsies (91 patients, 14.9%), a final cohort of 445 patients (73%) was selected for analysis. This final cohort constituted the study group for the present report.

### Patient characteristics

The main characteristics of the analyzed patients are presented in **Table 1**. The median patient age was 37.3 years, with a median proteinuria of 2.8 g/day and a median eGFR of 87.5 mL/min/1.73 m². Females predominated (82%), with ISN/RPS Class III/III+V in 20.7% of cases and Class IV/IV+V in 49.2%, without statistically significant differences between the two groups (**Table 2**).

**Table 1:** Baseline patient characteristics at the time of native kidney biopsy grouped by isolated C3 serum levels at 6 month (i-LowC3) follow-up (quantitative variables).

Variable	All (445 patients)					i-LowC3 (91 patients)					Controls (354 patients)					
	Percentiles					Percentiles					Percentiles					P Value
	**10**	**25**	**50**	**75**	**90**	**10**	**25**	**50**	**75**	**90**	**10**	**25**	**50**	**75**	**90**	
**C3 (mg/dL)**	28.3	44.7	**65.0**	86.0	111.8	29.0	40.0	**56.0**	75.0	85.2	27.3	45.0	**69.0**	90.0	115.0	**0.001**
**C4 (mg/dL)**	3.0	6.0	**10.7**	18.0	27.0	5.0	8.0	**12.0**	15.0	22.0	3.0	5.0	**10.0**	19.0	30.0	**0.048**
**Body weight (Kg)**	50.2	56.0	**63.5**	73.6	82.7	50.0	55.0	**61.3**	72.8	80.0	51.0	56.0	**64.0**	74.0	85.0	0.256
**Age (years)**	20.4	27.0	**37.3**	46.9	56.4	19.8	24.7	**34.6**	45.4	56.4	20.5	27.3	**37.9**	47.0	56.5	0.145
**Creatinine (mg/dL)**	0.6	0.7	**0.9**	1.3	2.1	0.6	0.8	**1.0**	1.6	2.5	0.6	0.7	**0.9**	1.3	2.0	**0.007**
**eGFR (ml/min/1.73 m2)**	30.5	52.1	**87.5**	112.7	125.6	25.1	43.6	**76.1**	106.7	125.3	33.1	56.0	**90.1**	114.2	126.0	**0.014**
**Proteinuria (g/dL)**	0.8	1.4	**2.8**	5.0	8.8	0.8	1.5	**3.1**	5.6	10.9	0.8	1.4	**2.7**	5.0	7.7	0.174
**Follow-up (years)**	1.1	2.5	**4.9**	9.4	18.7	1.0	2.9	**4.9**	9.4	24.2	1.1	2.4	**4.9**	9.4	18.1	0.586
**Systolic BP (mmHg)**	110	120	**130**	140	150	110	120	**130**	150	158	110	120	**127**	140	150	0.075
**Diastolic BP (mmHg)**	65	70	**80**	87	94	70	70	**80**	90	100	65	70	**80**	85	90	**0.028**
**Hemoglobin (g/dL)**	9.0	10.0	**11.5**	12.6	13.6	8.5	9.9	**11.3**	12.2	13.4	9.0	10.0	**11.5**	12.6	13.6	0.304

P values of Mann-Whitney U Test for 2 independent samples. Bold values in the P Value column indicate that percentile distributions in the two groups are statistically different.

**Table 2:** Patient characteristics grouped by isolate Low C3 at 6 months follow-up (categorical variables).

Variable	All (445 patients)	i-LowC3 (91 patients)	Controls (354 patients)	P Value
Gender (F) (n/%)	365/82%	73/80.2%	292/82.5%	0.616
ISN/RPS Class (n/%)				0.145
I	11/2.5%	1/1.1%	10/2.9%	
II	34/7.6%	10/11.1%	24/6.9%	
III or III+V	92/20.7%	14/15.6%	78/22.3%	
IV or IV+V	219/49.2%	52/57.8%	167/47.7%	
V	84/18.9%	13/14.4%	71/20.3%	
Missing	5/1.1%	1/1.1%	4/1.1%	

P values from Chi square test. The distribution of patients by gender or ISN/RPS Class was not different in the isolated Low C3 (i-LowC3) group compared to the Others.

As expected, the median pre-biopsy levels of C3 and C4 in the study population were low (65 mg/dL and 10.7 mg/dL, respectively) (**Table 1**). Among the cohort, 91 patients (20.4%) were identified as belonging to the i-LowC3 group. Age (**Table 1**) and gender (**Table 2**) distributions were similar between the i-LowC3 and control groups.

C3 serum levels before biopsy were significantly lower in the i-LowC3 group compared with the control group (56.0 mg/dL vs. 69.0 mg/dL, P = 0.001). Additionally, the i-LowC3 group compared with the control group demonstrated significantly reduced renal function at baseline (median eGFR of 76.1 vs. 90.1 mL/min/1.73 m², P = 0.014, **Table 1**). The i-LowC3 group, at baseline shows higher diastolic blood pressure values (P = 0.028) compared with the control group (**Table 1**).

### Immunosuppressive treatments

The use of immunosuppressive agents prescribed after renal biopsy is detailed in **Table 3**. Mycophenolate mofetil (MMF) (59.6%) and cyclophosphamide (31.2%) were the most frequently administered drugs, in addition to corticosteroids and Hydroxychloroquine. However, the distribution of LN treatments differed significantly between the two groups; immunosuppressive agents, particularly MMF, were less frequently used in the i-LowC3 group. MMF use was also less frequent in non-proliferative classes 1 and 2 compared to proliferative classes 3 and 4 (55.0% vs. 61.7%), though this difference was not statistically significant (P = 0.192). A trend toward reduced Hydroxychloroquine use in the i-LowC3 group was also noted (**Table 3**).

**Table 3:** Immunosuppressive therapies grouped by i-LowC3.

Variable	All (445 patients) n (%)	i-LowC3 (91 patients) n (%)	Controls (354 patients) n (%)	P Value
Corticosteroids	424 (95.3)	86 (94.5)	338 (95.5)	0.696
Hydroxychloroquine	258 (58.4)	45 (49.5)	213 (60.2)	0.053
Immunosuppressive treatments	391 (87.9)	72 (79.1)	319 (90.1)	**0.004**
N. of Immunosuppressive agents*				**0.044**
0	44 (9.9)	29 (8.2)	15 (16.5)	
1	251 (56.4)	209 (59.0)	42 (46.2)	
2	127 (28.5)	97 (27.4)	30 (33.0)	
3	23 (5.2)	19 (5.4)	4 (4.4)	
Mycophenolate	265 (59.6)	42 (46.2)	223 (63.0)	**0.004**
Cyclophosphamide	139 (31.2)	29 (31.9)	110 (31.1)	0.884
Rituximab	60 (13.5)	15 (16.5)	45 (12.7)	0.347
Azathioprine	54 (12.1)	12 (13.2)	42 (11.9)	0.730
Cyclosporin A	26 (5.8)	7 (7.7)	19 (5.4)	0.399
Tacrolimus	10 (2.3)	2 (2.2)	8 (2.3)	0.963
PlasmaExchange	9 (2)	3 (3.3)	6 (1.7)	0.333
Chlorambucil	2 (0.4)	1 (1.1)	1 (0.3)	0.299

P values from Chi square test. Bold values in the P Value column indicate that percentages in the two groups are statistically different.

### Serum complement levels trends

After the first six months of follow-up, a median increase of 20 mg/dL in C3 levels was observed, accompanied by a similar pattern for C4, with a median increase of 5 mg/dL. Correlation coefficients for C3 and C4 levels were 0.72 at baseline and 0.63 at six months, both statistically different from the zero value (P < 0.001).

### Outcome results

The study outcomes, grouped by i-LowC3 and normal C4 levels at the six-month follow-up, are presented in **Table 4**. CKD, the composite outcome of CKD or death, ESKD, and the composite outcome of ESKD or death were all significantly directly associated with i-LowC3 (P < 0.001). The risk of CKD in patients with i-LowC3 compared with the control group was more than double, increasing from 17.8% to 40.7% (228%).

**Table 4:** Patient outcomes grouped by i-LowC3.

Variable	All (445 patients) n (%)	i-LowC3 (91 patients) n (%)	Controls (354 patients) n (%)	P Value
CKD	100 (22.5)	37 (40.7)	63 (17.8)	**<0.001**
CKD or Death	104 (23.4)	37 (40.7)	67 (18.9)	**<0.001**
ESKD	25 (5.6)	15 (16.5)	10 (2.8)	**<0.001**
ESKD or Death	34 (7.6)	18 (19.8)	16 (4.5)	**<0.001**

CKD and CKD or Death were both associated with i-LowC3. Bold values means P values from Chi square test.

### Univariate survival analysis

**Figure 1:**
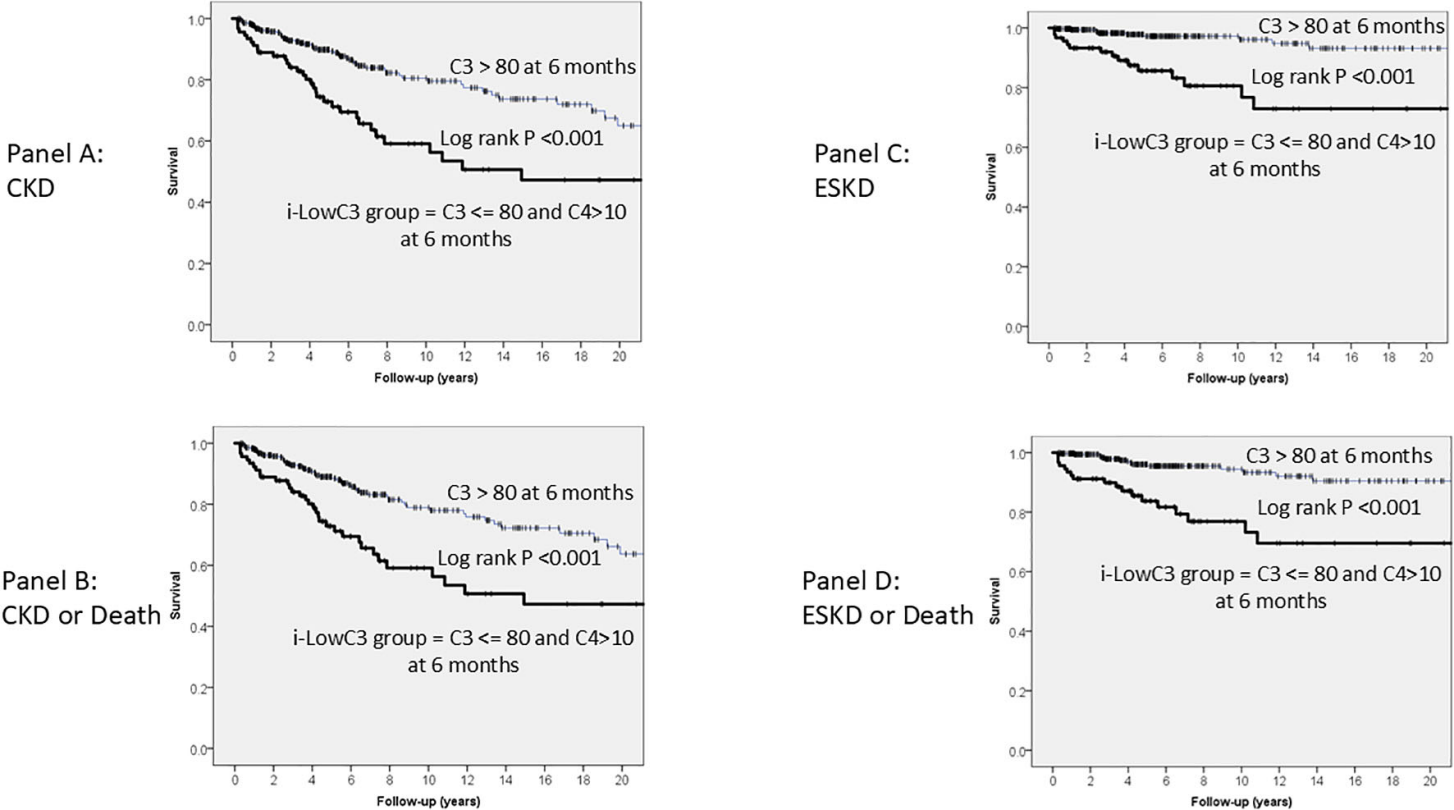
Kaplan-Meier long-term survival curves for CKD **(A)**, CKD or Death **(B)**, ESKD **(C)** and ESKD or Death **(D)**, grouped, at six month follow up, by levels of C3 <= 80 mg/dL and C4 > 10 mg/dL (i-LowC3 group). In all cases, survival was lower in the i-LowC3 group compared with Controls.

Kaplan-Meier long-term survival curves for CKD Panel A), the composite outcome of CKD or death (Panel B), ESKD (Panel C) and the composite outcome ESKD or death (Panel D) are shown in **Figure 1**. In all cases, survival was significantly lower in the i-LowC3 group at the six-month follow-up compared to the control group (Log-rank test, P < 0.001 for all Panels).

### Multivariate Cox regression analysis

Multivariate Cox regression analyses of predictors associated with CKD (Panel A) and the composite outcome of CKD or death (Panel B) are shown in **Table 5A**. A lower risk of CKD and CKD or death was significantly associated with: (i) higher eGFR before biopsy; (ii) a decrease in proteinuria at the six-month follow-up, and (iii) an increase in C3 levels at the six-month follow-up compared with their baseline values at the time of biopsy. Conversely, a higher risk of CKD and CKD or death was significantly associated with: (i) i-LowC3 group, (ii) higher proteinuria before biopsy, (iii) older age at biopsy, and (iiii) MMF treatment.

**Table 5a:** Multivariate Cox regression analysis of the predictors associated with CKD (Panel A) and CKD or Death (Panel B).

Panel A: CKD							
Variable	B	SE	Wald	P value	RR	RR 95% CI	
i-LowC3 (Yes)	0.493	0.237	4.318	0.038	1.637	1.028	2.605
Proteinuria before biopsy (g/dL)	0.112	0.034	10.677	0.001	1.118	1.046	1.196
Proteinuria decrease at six months (g/day)	-0.092	0.039	5.613	0.018	0.912	0.845	0.984
eGFR before biopsy (ml/min/1.73 m2)	-0.026	0.004	53.312	<0.001	0.974	0.967	0.981
Age at biopsy (years)	0.017	0.008	5.090	0.024	1.017	1.002	1.033
Mycophenolate (Yes)	0.836	0.255	10.758	0.001	2.307	1.400	3.801
Serum C3 increase at six months (mg/dL)	-0.012	0.004	10.188	0.001	0.988	0.980	0.995

Panel B: CKD or Death							
Variable	B	SE	Wald	P value	RR	RR 95% CI	
i-LowC3 (Yes)	0.424	0.235	3.251	0.071	1.527	0.964	2.420
Proteinuria before biopsy (g/dL)	0.108	0.034	10.286	0.001	1.115	1.043	1.191
Proteinuria decrease at six months (g/day)	-0.086	0.038	5.140	0.023	0.917	0.851	0.988
eGFR before biopsy (ml/min/1.73 m2)	-0.026	0.004	52.836	<0.001	0.975	0.968	0.982
Age at biopsy (years)	0.020	0.007	6.823	0.009	1.020	1.005	1.035
Micofenolate (Yes)	0.765	0.248	9.529	0.002	2.148	1.322	3.490
C3 increase at six months (mg/dL)	-0.012	0.004	10.021	0.002	0.988	0.981	0.995

Panel A: A major risk CKD was associated with the isolated Low C3 group at six month follow up, high values of proteinuria before biopsy, a major age at biopsy and the therapy with Mycophenolate, while a minor risk was associated with a decrease of proteinuria at six months of follow up, a higher eGFR before biopsy and an increase of C3 levels at six months of follow up.

Panel B: A major risk CKD or death was associated with higher values of proteinuria before biopsy, a major age at biopsy and with the therapy with Mycophenolate, while a minor risk was associated with a decrease of proteinuria at six months of follow up, a higher eGFR before biopsy and an increase of C3 levels at six months of follow up. The isolated Low C3 group at six month follow up was associated with a poor renal prognosis (RR 1.527) with a border line statistical significance (P=0.071).

B: beta coefficient; SE: Standard Error of beta coefficient,; Wald: Wald statistic; RR: Relative Risk; RR 95% CI: 95% confidence intervals of RR.

**Table 5b:** Multivariate Cox regression analysis of the predictors associated with ESKD (Panel A) and ESKD or death (Panel .

Panel A: ESKD							
Variable	B	SE	Wald	P value	RR	RR 95% CI	
C3 before biopsy (mg/dL)	0.022	0.007	9.716	0.002	1.022	1.008	1.036
i-LowC3 (Yes)	1.397	0.475	8.630	0.003	4.042	1.592	10.264
Baseline eGFR (ml/min/1.73 m2)	-0.033	0.008	16.449	<0.001	0.968	0.952	0.983
Age at biopsy (years)	-0.063	0.022	7.958	0.005	0.939	0.899	0.981
Hydroxychloroquine (Yes)	-1.391	0.775	3.224	0.073	0.249	0.054	1.136
Panel B: ESKD or Death							
Variable	B	SE	Wald	P value	RR	RR 95% CI	
C3 before biopsy (mg/dL)	0.022	0.006	13.592	<0.001	1.022	1.010	1.035
i-Low C3 (Yes)	1.539	0.395	15.179	<0.001	4.662	2.149	10.113
Baseline eGFR (ml/min/1.73 m2)	-0.023	0.007	12.677	<0.001	0.977	0.965	0.990
PlasmaExchange (Yes)	1.386	0.644	4.636	0.031	3.999	1.132	14.121
Hydroxychloroquine (Yes)	-1.191	0.561	4.502	0.034	0.304	0.101	0.913

Panel A: A major risk of ESKD was associated with high levels of C3 before biopsy and with i-LowC3 levels at six month follow up, while a minor risk was associated with a higher eGFR before biopsy, with higher age at biopsy and with a therapy with Hydroxychloroquine.

Panel B: A major risk of ESKD or death was associated with high levels of C3 before biopsy, with i-LowC3 and with the treatment with plasmaexchange, while a minor risk was associated with a higher eGFR before biopsy and with the therapy with Hydroxychloroquine.

B: beta coefficient; SE: Standard Error of beta coefficient; Wald: Wald statistic; RR: Relative Risk; RR 95% CI: 95% confidence intervals of RR.

Predictors associated with ESKD (Panel A) and with the composite outcome of ESKD or death (Panel B) are shown in **Table 5B**. A major risk of ESKD was associated with high levels of C3 before biopsy and with i-LowC3 levels at six month follow up, while a minor risk was associated with a higher eGFR before biopsy, with higher age at biopsy and with a therapy with Hydroxychloroquine (Panel A). A major risk of ESKD or death was associated with high levels of C3 before biopsy and i-LowC3 at six month follow up and with the treatment with plasmaexchange, while a minor risk was associated with a higher eGFR before biopsy and with the therapy with Hydroxychloroquine (Panel B).

The proliferative histological classes (Class 3 and Class 4) compared to non-proliferative classes (Class 1 and Class 2) were not significantly associated with the risk of CKD (P = 0.585) or CKD or death (P = 0.804). Additionally, the interaction of MMF therapy with proliferative (Classes 3 and 4) versus non-proliferative (Classes 1, 2 and 5) histological classes was not significant for CKD (P = 0.377) or CKD or death (P = 0.356). This indicates that the increased risk of CKD and CKD or death associated with MMF exposure was present in both proliferative and non-proliferative histological classes.

## Discussion

This large, retrospective, multicenter study from the Italian Registry of Renal Biopsy (IRRB), conducted in patients with a newly established histological diagnosis of LN, provides robust evidence that i-LowC3, a low-cost and readily available biomarker, is strongly associated with an increased doubled risk of CKD, ESKD or death. This predictive effect persisted after adjusting for initial disparities in eGFR between groups, as well as other confounding variables, including 24-hour proteinuria and induction treatment regimens (**Tables 5A, B**). The survival curves demonstrate the worse prognosis of the i-LowC3 group, across all considered endpoints. Furthermore, our results suggest that an increase in serum C3 levels at six months confers additional significant protection against the development of CKD (1% lower risk for each mg increase of C3, **Table 5A**).

The findings of this study align with and extend the results of a recent retrospective investigation (18), which assessed the renal prognosis of an international cohort of patients with LN. That study stratified patients based on the presence of persistent i-LowC3, hypothesizing that the persistent activation of the cAP - reflected by persistently reduced serum C3 levels (following adequate induction treatment) - represents a failure to suppress complement-mediated kidney injury. This earlier investigation demonstrated that patients with LN and persistent i-LowC3 exhibited a markedly increased risk of CKD, even when controlling for established prognostic factors such as age, sex, ethnicity and baseline eGFR.

Similarly, our study analyzed complement dynamics over six months following the first renal biopsy in a large cohort of LN patients, encompassing all histological classes. These results further support the hypothesis that serum C3 and C4 levels post-immunosuppressive therapy function as independent predictors of renal outcomes. This interpretation is consistent with experimental findings demonstrating the critical role of complement factor H (CFH), a key regulator of the cAP, in mitigating kidney injury. For instance, lupus-prone mice deficient in CFH exhibit unregulated cAP activation, hypocomplementemia, severe proteinuria, accelerated kidney failure, and early mortality compared to CFH-intact counterparts (23). Conversely, experimental deletion of other essential cAP components, such as complement factor B (24) or factor D (25), significantly reduces renal injury severity in murine lupus models.

Despite these experimental insights, translating these findings to clinical practice has proven challenging. While the association between low serum C3 levels and LN (26) and between complement levels normalization and favorable renal outcomes in LN (27) are well-established, and transient reductions in serum C3 and/or C4 levels are frequently associated with renal flares (15), these observations have not been sufficient to reliably identify individual patients at high risk for CKD. Furthermore, prior studies often assessed low serum C3 levels without concurrently analyzing fluctuations in C4 levels, thereby conflating the pathological effects of persistent cAP dysregulation (as reflected by i-LowC3) with the physiological activation of the cCP due to circulating immune complexes (reflected by reductions in both C3 and C4). This conflation has obscured the specific contribution of cAP dysregulation to LN-related kidney damage.

Recent studies are beginning to clarify these complex interactions. For instance, Song et al. demonstrated that serum Bb, a cAP activation product derived from complement factor B, is elevated during LN flares and is a significant predictor of poor renal outcomes (20). Furthermore, other investigations have identified anti-C3 autoantibodies that stabilize the cAP C3 convertase, thereby perpetuating cAP dysregulation and resulting in persistent C3 consumption (19). These findings provide a mechanistic basis for the observed association between persistent low serum C3 levels and adverse renal outcomes.

In addition, this study underscores the clinical utility of monitoring trends in serum complement levels, particularly i-LowC3, as an independent predictor of CKD and ESKD in LN. These results contribute to the growing body of evidence supporting the role of complement in LN pathogenesis and highlight the potential of complement-targeted therapies to address the unmet needs of this high-risk patient subgroup.

The clearer delineation of the roles of complement cascade activation pathways in LN provides critical insights, particularly regarding therapeutic considerations at the end of the initial period of immunosuppressive therapy. Persistent activation of the cAP appears to be a key driver of poor renal prognosis. While standard LN therapies, such as cyclophosphamide or MMF, may effectively control the proliferative inflammatory component, failure to rapidly correct cAP dysregulation could promote progressive endothelial damage, ultimately leading to CKD and ESKD. It is interesting to note that a similar pathophysiological role of a defective complement regulation has been suggested also in active and progressive cases of immunoglobulin A nephropathy (28–30).

In light of the findings by Vasilev et al. (19), therapies that achieve profound and sustained suppression of antibody production may facilitate a more rapid restoration of cAP balance, taking also into account that likely antiC3-autoantibodies and C3 Nephritic factor have different pathway actions. It is noteworthy that studies evaluating the addition of anti-CD20 therapies (rituximab, obinutuzumab) to standard LN care demonstrated faster normalization of serum C3 levels in treated patients compared with the controls. However, the follow-up durations in these studies were insufficient to confirm whether normalizing serum C3 translates into renal protection and prevention of ESKD (31–35). Preliminary data suggest that obinutuzumab, in particular, may more effectively rebalance the cAP and mitigate the risk of ESKD (36).

Our study also analyzed follow-up data on proteinuria and immunosuppressive treatments. In alignment with prior studies, our results reaffirm that 24-hour proteinuria serves as a key predictor of renal outcomes: higher proteinuria at baseline predicted worse outcomes, while a significant reduction after six months of induction therapy was associated with favorable renal prognosis. Additionally, our findings underscore the nephroprotective role of Hydroxychloroquine.

### Limitations of the study

The major limitations of this study include the retrospective design and the related difficulties in the interpretations of some results. Notably, patients in the i-LowC3 group received significantly less immunosuppressive therapy and particularly less cyclophosphamide (**Table 3**), a finding that is challenging to interpret given their significantly lower eGFR at the time of biopsy—a negative prognostic marker that would typically warrant more aggressive therapy. In addition, treatment differences could be due to center-specific and time-varying protocols, toxicity concerns or delayed diagnosis, that are unavailable in this study.

Despite this limitation, the strong association between i-LowC3 and CKD risk remained significant even after adjusting for baseline eGFR and prescribed therapies (**Tables 5A, B**). Another surprising aspect comes from the analysis of used immunosuppressive therapies. In particular, we observed an elevated risk of CKD and ESKD for patients exposed to Mycophenolate (MMF) treatment. We know that the fixed MMF dosing used in clinical practice and trials may not provide adequate levels of the active MMF metabolite mycophenolic acid (MPA). Up to tenfold variation in the MPA area under the plasma concentration-time curve has been observed with fixed MMF dosing (37). This variability could consequently have led to a negative effect on the renal outcome over longer follow-up times than in conventional clinical trials. It is interesting to note that a systematic review evaluating the risk of ESKD in LN patients in published studies from 1971 to 2015 identified an increase of the ESKD risk probably due to the progressive substitution of cyclophosphamide with MMF (38). Our study seems to confirm that MMF is related to CKD/ESKD risk, however, due to the retrospective design of the study, it was not possible to account for all confounding factors influencing treatment decisions during the induction phase. For instance, patients treated with MMF could be negatively selected, when their clinical evaluation suggested poor renal prognosis and selection treatment bias of sicker patients preferentially receiving MMF.

## Conclusions

This study suggests that, six months after the renal biopsy: 1) The combination of serum C3 levels below normal with normal C4 levels, once inflammatory activity is controlled with immunosuppression, identifies LN patients at high risk of developing CKD and ESKD, and 2) Linear increases in C3 levels reduce independently the same risk. Isolated low C3 at 6 month follow up was associated with twice the risk of CKD, ESKD or death, highlighting the plausible critical role of cAP activation as a driver of kidney damage. These findings underscore the need for further investigations into complement-targeted treatments in LN, particularly in patients with i-LowC3.

## Data Availability

Data supporting this study may be shared upon reasonable request to the corresponding author.

## Appendix: Cities, affiliations and names of collaborative authors of the ITA-KID-BIOPSY Group.

**Table:**

ACIREALE, Ospedale Santa Marta e Santa Venera:	Maurizio Garozzo, Giovanni Giorgio Battaglia
AGRIGENTO, U.O.C Nefrologia e Dialisi San Giovanni di Dio:	Antonio Granata, Giulio Distefano, Monica Insalaco, Rosario Maccarrone
ALBANO LAZIALE, Ospedale Regina Apostolorum:	Marco Leoni, Angelo Emanuele Catucci, Emanuela Cavallaro
ALESSANDRIA, AOU “SS Antonio e Biagio e Cesare Arrigo”:	Marco Quaglia, Brigida Brezzi
ANCONA, Umberto I:	Carolina Finale, Valentina Nastasi, Andrea Ranghino, Domenica Taruscia
AOSTA, Ospedale Regionale Umberto Parini:	Massimo Manes, Andrea Molino
ASCOLI PICENO, Ospedale “C. e G. Mazzoni”:	Giuseppe Fioravanti, Cinzia Fiori, Rosaria Polci
ASTI, S.C. Nefrologia e Dialisi Osp. Cardinal Massaia:	Olga Randone, Nicola Giotta, Stefano Maffei
BARI, Policlinico - Ist. Nefrologia:	Loreto Gesualdo, Annamaria Di Palma, Michele Rossini, Adele Mitrotti, Diletta Stea, Adriano Montinaro
BARI, UOSD Nefrologia e dialisi pediatrica Ospedale Giovanni XXIII	Diletta Domenica Torres, Sebastiano Nestola, Mario Giordano, Donatella Simone
BOLOGNA, IRRCS Azienda Ospedaliero-Universitaria, Nephrology, Dialysis and Kidney Transplant Unit, Sant’Orsola Hospital:	Gaetano La Manna, Olga Baraldi, Claudia Bini, Gisella Vischini
BORGOMANERO, Osp. SS. Trinità:	Luisa Benozzi, Stefano Cusinato
BRINDISI, Ospedale A. Perrino:	Luigi Vernaglione, Brigida Di Renzo, Cosima Balestra, Palmira Schiavone, Alessio Montanaro, Concetta Giovine
CASTELFRANCO VENETO, Osp. S. Giacomo:	Abaterusso Cataldo, Roberta Lazzarin
CATANIA, Nefrologia Cannizzaro:	Antonio Granata, Giuseppe Seminara, Daniela Puliatti
CHIETI, Policlinico “SS. Annunziata”:	Mario Bonomini, Roberto Di Vito, Teresa Lombardi, Luigia Larlori
CIRIÈ, Ospedale Civile:	Carolina Maria Licata, Vincenza Calitri
COMO, S. Anna:	Marco D’Amico, Beniamina Gallelli
CUNEO, Osp. S. Croce:	Elisabetta Moggia
EBOLI, Ospedale Maria Santissima Addolorata:	Giuseppe Gigliotti, Pierluigi D’Angiò, Simona Laurino, Antonello Mancini
FERRARA, S. Anna:	Yuri Battaglia, Alda Storari, Sergio Sartori, Paola Tombesi
FOGGIA, Nefrologia Universitaria:	Barbara Infante, Giulia Godeas, Giovanni Stallone
FORLÌ, Morgani Pierantoni:	Fulvia Zanchelli, Giovanni Mosconi
GENOVA, UO Nefrologia, Dialisi e Trapianto. IRCCS Policlinico San Martino	Francesca Viazzi, Pasquale Esposito, Margherita Di Martino
LA SPEZIA, Sant’Andrea:	Lucio Manenti, Valentina Corbani, Francesca Lauria, Laura Panaro, Francesca Cappadona
LECCO, Associazione Italiana Ricercare per Curare ODV ETS (AIRpC):	Simeone Andrulli, Giovanni De Vito, Salvatore David e Giovanni Valsecchi
MESSINA, Policlinico-Nefrologia:	Domenico Santoro, Chiara Casuscelli, Guido Gembillo, Antonello Salvo
MILANO, ASST Fatebenefratelli-Sacco, Università di Milano:	Maurizio Gallieni, Sabrina Caruso, Nicoletta Landriani, Gianmarco Sabiu, Daniela Alfano
MILANO, Humanitas	Gabriella Moroni, Francesco Reggiani, Marta Calatroni, Emanuele Conte, Claudio Angelini
MODENA, Policlinico:	Francesco Fontana, Riccardo Magistroni, Ronni Luca Spaggiari, Francesca Testa, Gianni Cappelli
NAPOLI, Università della Campania - Nefrologia:	Giovambattista Capasso, Rosa Maria Pollastro, Davide Viggiano
NOVARA, Azienda Ospedaliero-Universitaria Maggiore della Carità:	Doriana Chiarinotti, Maria Maddalena Conte
NOVARA, Nephrology and Transplant Unit, “Maggiore della Carità” University Hospital, Università del Piemonte Orientale:	Marco Quaglia
NUORO, Ospedale San Francesco	Giovanni Mattana, Marilena Sedda, Anna Orru, Antonio Pais, Annalisa Sini, Agostina Angela Leoni, Francesco Logias
PALERMO, Ospedale Cervello:	Angelo Ferrantelli, Maria Giovanna Vario
PALERMO, UO di Nefrologia Dialisi e Trapianto ARNAS Civico:	Gioacchino Li Cavoli, Luisa Bono, Antonio Amato
PARMA, Nephrology Unit, University Hospital	Lucio Manenti, Isabella Pisani, Monia Incerti
PERUGIA, Ospedale Santa Maria della Misericordia:	Rachele Brugnano, Raffaela Sciri, Ilaria Carriero
PESARO, Ospedali Riuniti Marche Nord:	Marina Di Luca, Flavia Manenti, Marco Palladino, Xhensila Gabrocka, Francesca Pizzolante
PISA, S. Chiara-Nefrologia:	Antonio Pasquariello, Giovanna Pasquariello, Maurizio Innocenti, Matilde Masini
PISA, U.O. Nefrologia Dia lisi e Trapianti, Osp. CISANELLO:	Domenico Giannese
RAVENNA, S. Maria delle Croci:	Andrea Buscaroli, Mattia Monti
REGGIO EMILIA, S. Maria Nuova:	Enrica Gintoli, Elisa Gnappi, Mariacristina Gregorini
RIMINI, Osp. Degli Infermi:	Paola De Giovanni, Angelo Rigotti
ROMA, Policl. Umberto I:	Rosario Cianci, Antonietta Gigante
ROMA, UOC di Nefrologia, Policlinico Agostino Gemelli	Francesca Bruno, Giuseppe Grandaliano
S. GIOVANNI ROTONDO, Fondazione Casa Sollievo della Sofferenza IRCCS:	Filippo Aucella, Michele Antonio Prencipe, Aurora Perez Ys, Francesco Aucella
TARANTO, SS. Annunziata:	Luigi Morrone, Arcangelo Di Maggio
TORINO, Osp. Mauriziano:	Cristina Marcuccio
TORINO, Ospedale Martini:	Roberto Boero, Giulio Cesano
TRENTO, Presidio Ospedaliero Santa Chiara:	Laura Sottini, Giuliano Brunori
VERBANIA, Osp. Castelli:	Maurizio Borzumati, Maria Carmela Vella, Stefania Gioira
VERCELLI, S. Andrea:	Giovanna Mele, Luigia Costantini, Patrizia Colombo
VITERBO, Belcolle:	Corrado Murtas, Roberta Guastini, Franca Luchetta, Rossella Iacono, Emanuela Cristi, Teresa Valentina Ranalli, Sandro Feriozzi
